# 2-(Piperidin-1-yl)-6-(1*H*-pyrrol-1-yl)pyridine-3,5-dicarbonitrile

**DOI:** 10.1107/S1600536812008586

**Published:** 2012-03-03

**Authors:** Peter N. Horton, Shaaban K. Mohamed, Ahmed M. Soliman, Eman M. M. Abdel-Raheem, Mehmet Akkurt

**Affiliations:** a School of Chemistry, University of Southampton, Highfield, Southampton SO17 1BJ, England; b Chemistry and Environmental Science Division, School of Science, Manchester Metropolitan University, England; c Department of Chemistry, Faculty of Science, Sohag University, Sohag, Egypt; d Department of Physics, Faculty of Sciences, Erciyes University, 38039 Kayseri, Turkey

## Abstract

The piperidine ring of the title compound, C_16_H_15_N_5_, adopts a chair conformation. The pyridine ring is essentially planar, with a maximum deviation of 0.035 (3) Å. The pyrrole and pyridine rings are almost coplanar, forming a dihedral angle of 3.48 (14)°. In the crystal, no classical hydrogen bonds were found. In the crystal, the molecules are linked by aromatic π–π stacking [centroid–centroid separations = 3.4984 (16) and 3.9641 (15) Å between pyrrole and pyridine rings and between pyridine rings, respectively].

## Related literature

For the biological activity of cyano-amino pyridines, see: Al-Haiza *et al.* (2003 ▶); Bhalerao & Krishnaiah (1995 ▶); Doe *et al.* (1990 ▶); Murata *et al.* (2003 ▶); Shankaraiah *et al.* (2010 ▶); Shishoo *et al.* (1983 ▶); Soliman *et al.* (2012 ▶); Temple *et al.* (1992 ▶). For ring conformations, see: Cremer & Pople (1975 ▶).
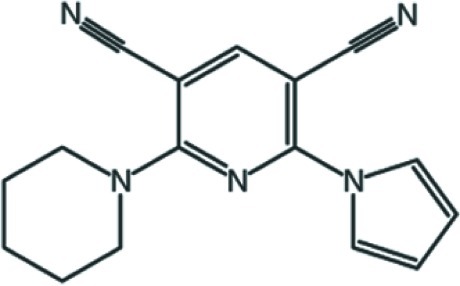


## Experimental

### 

#### Crystal data

C_16_H_15_N_5_
*M* *_r_* = 277.33
Monoclinic, 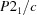

*a* = 11.9372 (16) Å
*b* = 6.6919 (8) Å
*c* = 17.158 (2) Å
β = 92.280 (7)°
*V* = 1369.5 (3) Å^3^
*Z* = 4
Mo *K*α radiation
μ = 0.09 mm^−1^
*T* = 100 K
0.32 × 0.04 × 0.02 mm

#### Data collection

Rigaku Saturn724+ diffractometer
Absorption correction: multi-scan (*CrystalClear-SM Expert*; Rigaku, 2011 ▶) *T*_min_ = 0.973, *T*_max_ = 0.998
7877 measured reflections
3098 independent reflections
1503 reflections with *I* > 2σ(*I*)
*R*_int_ = 0.095

#### Refinement

*R*[*F* ^2^ > 2σ(*F* ^2^)] = 0.073
*wR*(*F* ^2^) = 0.140
*S* = 0.96
3098 reflections
190 parameters
H-atom parameters constrained
Δρ_max_ = 0.21 e Å^−3^
Δρ_min_ = −0.24 e Å^−3^

### 

Data collection: *CrystalClear-SM Expert* (Rigaku, 2011 ▶); cell refinement: *CrystalClear-SM Expert*; data reduction: *CrystalClear-SM Expert*; program(s) used to solve structure: *SIR2004* (Altomare *et al.*, 1999 ▶); program(s) used to refine structure: *SHELXL97* (Sheldrick, 2008 ▶); molecular graphics: *ORTEP-3 for Windows* (Farrugia, 1997 ▶) and *PLATON* (Spek, 2009 ▶); software used to prepare material for publication: *WinGX* (Farrugia, 1999 ▶) and *PLATON*.

## Supplementary Material

Crystal structure: contains datablock(s) global, I. DOI: 10.1107/S1600536812008586/xu5473sup1.cif

Structure factors: contains datablock(s) I. DOI: 10.1107/S1600536812008586/xu5473Isup2.hkl

Supplementary material file. DOI: 10.1107/S1600536812008586/xu5473Isup3.cml

Additional supplementary materials:  crystallographic information; 3D view; checkCIF report

## Figures and Tables

**Fig. 1.**
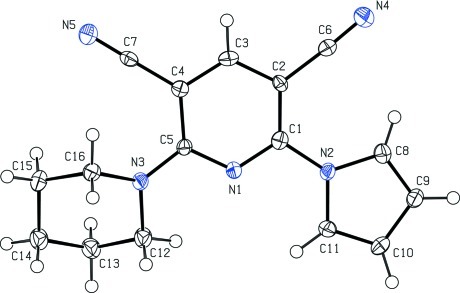
The molecular structure of (I), showing the atom-numbering scheme and displacement ellipsoids drawn at the 50% probability level.

**Fig. 2.**
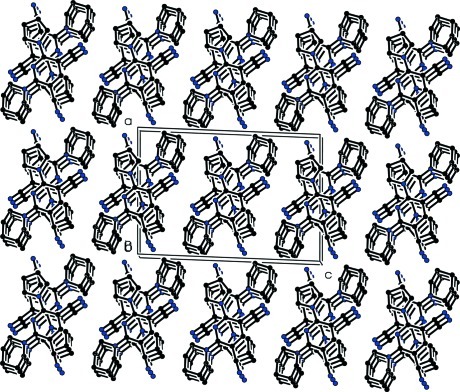
Packing of (I) down the b axis. The hydrogen atoms have been omitted for clarity.

## supplementary crystallographic information

### Comment

Among the wide variety of active heterocycles, cyano-amino pyridines have showed important and useful intermediates in preparing variety of heterocyclic compounds (Shishoo *et al.*, 1983; Doe *et al.*, 1990; Bhalerao & Krishnaiah, 1995; Al-Haiza *et al.*, 2003). In addition to this, many naturally occurring and synthetic compounds containing the pyridine scaffold possess interesting pharmacological properties (Temple *et al.*, 1992). Among them, 2-amino-3-cyanopyridines have been identified as IKK-β inhibitors (Murata *et al.*, 2003) and as antibacterial (Shankaraiah *et al.*, 2010). Therefore, the synthesis of 2-amino-3-cyanopyridine derivatives continues to attract much interest in organic chemistry. In this respect, and also in continuation of our earlier work on synthesis of different heterocyclic system that containing highly biological activity (Soliman *et al.*, 2012), we prompted to prepare the new title compound (I) with potential biological activity.

Fig. 1 shows the molecule of (I) which has an open conformation. The N3/C12–C16 piperidine ring adopts a chair conformation [puckering parameters (Cremer & Pople, 1975): Q_T_ = 0.574 (3) Å, θ = 179.5 (3) ° and φ = 137 (13) °]. The N1/C1–C5 pyridine ring is essentially planar with a maximum deviation of -0.035 (3) Å for C5. The N2/C8–C11 pyrrole and pyridine rings are almost co-planar and they make a dihedral angle of 3.48 (14)° with each other.

The structure exists no classic hydrogen bonds. The crystal packing exhibits π—π interactions with centroid—centroid distances: *Cg*1—*Cg*2^i^ = 3.4984 (16) Å and *Cg*2—*Cg*2^ii^ = 3.9641 (15) Å [Fig. 2; *Cg*1 and *Cg*2 are the centroids of the N2/C8–C11 pyrrole and N1/C1–C5 pyridine rings, respectively. Symmetry codes: (i) 1 - *x*, 1 - *y*, -*z* and (ii) 1 - *x*, -*y*, -*z*].

### Experimental

An equimolar mixture of 2-chloro-6-(1*H*-pyrrol-1-yl) pyridine-3,5-dicarbonitrile and piperidine in THF/EtOH (1:3) with few drops of TEA was refluxed at 351 K for 2–3 h. The product was obtained on cooling, collected, washed and re-crystallized from ethanol to afford the title compound. 90% yield, m.p. 413 K. Block-like pure crystals of the title compound, suitable for X-ray diffraction, were obtained by slow evaporation of a solution in ethanol for 24 h.

### Refinement

All H atoms were positioned geometrically and refined as riding on their parent atoms with C—H distances of 0.93 Å and 0.97 Å. Isotropic displacement parameters for these atoms were set to 1.2 (CH, CH_2_) times *U*_eq_ of the parent atom. The (1 3 10) and (-4 6 14) reflections were omitted owing to bad disagreement. The *ADDSYM* routine in *PLATON* (Spek, 2009) suggests the space group *P2_1_/c* which is consistent with the *P2_1_/c* assignment of our structure.

### Crystal data

**Table:**

C_16_H_15_N_5_	*F*(000) = 584
*M**_r_* = 277.33	*D*_x_ = 1.345 Mg m^−^^3^
Monoclinic, *P*2_1_/*c*	Mo *K*α radiation, λ = 0.71073 Å
Hall symbol: -P 2ybc	Cell parameters from 4756 reflections
*a* = 11.9372 (16) Å	θ = 3.3–27.5°
*b* = 6.6919 (8) Å	µ = 0.09 mm^−^^1^
*c* = 17.158 (2) Å	*T* = 100 K
β = 92.280 (7)°	Lath, colourless
*V* = 1369.5 (3) Å^3^	0.32 × 0.04 × 0.02 mm
*Z* = 4	

### Data collection

**Table:**

Rigaku Saturn724+ diffractometer	3098 independent reflections
Radiation source: Rotating Anode	1503 reflections with *I* > 2σ(*I*)
Confocal monochromator	*R*_int_ = 0.095
Detector resolution: 28.5714 pixels mm^-1^	θ_max_ = 27.5°, θ_min_ = 3.3°
profile data from ω–scans	*h* = −15→15
Absorption correction: multi-scan (*CrystalClear-SM Expert*; Rigaku, 2011)	*k* = −7→8
*T*_min_ = 0.973, *T*_max_ = 0.998	*l* = −21→22
7877 measured reflections	

### Refinement

**Table:**

Refinement on *F*^2^	Primary atom site location: structure-invariant direct methods
Least-squares matrix: full	Secondary atom site location: difference Fourier map
*R*[*F*^2^ > 2σ(*F*^2^)] = 0.073	Hydrogen site location: inferred from neighbouring sites
*wR*(*F*^2^) = 0.140	H-atom parameters constrained
*S* = 0.96	*w* = 1/[σ^2^(*F*_o_^2^) + (0.0431*P*)^2^] where *P* = (*F*_o_^2^ + 2*F*_c_^2^)/3
3098 reflections	(Δ/σ)_max_ < 0.001
190 parameters	Δρ_max_ = 0.21 e Å^−^^3^
0 restraints	Δρ_min_ = −0.24 e Å^−^^3^

### Special details

**Table:**

Experimental. *CrystalClear-SM Expert*
Geometry. Bond distances, angles *etc*. have been calculated using the rounded fractional coordinates. All su's are estimated from the variances of the (full) variance-covariance matrix. The cell e.s.d.'s are taken into account in the estimation of distances, angles and torsion angles
Refinement. Refinement on *F*^2^ for ALL reflections except those flagged by the user for potential systematic errors. Weighted *R*-factors *wR* and all goodnesses of fit *S* are based on *F*^2^, conventional *R*-factors *R* are based on *F*, with *F* set to zero for negative *F*^2^. The observed criterion of *F*^2^ > σ(*F*^2^) is used only for calculating -*R*-factor-obs *etc*. and is not relevant to the choice of reflections for refinement. *R*-factors based on *F*^2^ are statistically about twice as large as those based on *F*, and *R*-factors based on ALL data will be even larger.

### Fractional atomic coordinates and isotropic or equivalent isotropic displacement parameters (Å^2^)

**Table:**

	*x*	*y*	*z*	*U*_iso_*/*U*_eq_	
N1	0.57393 (18)	0.2530 (3)	0.06283 (13)	0.0173 (7)	
N2	0.38710 (18)	0.2792 (3)	0.03066 (13)	0.0168 (7)	
N3	0.75463 (17)	0.2158 (3)	0.11176 (13)	0.0192 (7)	
N4	0.3876 (2)	0.2027 (4)	−0.19418 (14)	0.0312 (9)	
N5	0.9243 (2)	0.2228 (3)	−0.06919 (14)	0.0262 (8)	
C1	0.4966 (2)	0.2520 (4)	0.00532 (16)	0.0169 (9)	
C2	0.5227 (2)	0.2225 (4)	−0.07288 (15)	0.0150 (8)	
C3	0.6365 (2)	0.2034 (4)	−0.08826 (15)	0.0180 (9)	
C4	0.7185 (2)	0.2039 (4)	−0.02904 (15)	0.0167 (9)	
C5	0.6829 (2)	0.2212 (4)	0.04902 (15)	0.0153 (8)	
C6	0.4457 (2)	0.2118 (4)	−0.13900 (16)	0.0209 (9)	
C7	0.8327 (2)	0.2111 (4)	−0.05098 (15)	0.0174 (9)	
C8	0.2865 (2)	0.2905 (4)	−0.01382 (17)	0.0207 (9)	
C9	0.2014 (2)	0.3079 (4)	0.03534 (16)	0.0206 (9)	
C10	0.2483 (2)	0.3078 (4)	0.11287 (16)	0.0199 (9)	
C11	0.3609 (2)	0.2903 (4)	0.10890 (16)	0.0191 (9)	
C12	0.7216 (2)	0.2875 (4)	0.18847 (16)	0.0250 (9)	
C13	0.8159 (2)	0.4145 (4)	0.22429 (17)	0.0255 (10)	
C14	0.9264 (2)	0.3013 (4)	0.22895 (17)	0.0263 (10)	
C15	0.9552 (2)	0.2224 (4)	0.14853 (17)	0.0233 (9)	
C16	0.8582 (2)	0.0981 (4)	0.11522 (16)	0.0212 (9)	
H3	0.65750	0.19000	−0.13960	0.0220*	
H8	0.27940	0.28670	−0.06800	0.0250*	
H9	0.12570	0.31800	0.02100	0.0250*	
H10	0.20860	0.31800	0.15830	0.0240*	
H11	0.41200	0.28630	0.15120	0.0230*	
H12A	0.70700	0.17480	0.22220	0.0300*	
H12B	0.65350	0.36630	0.18270	0.0300*	
H13A	0.79650	0.45540	0.27630	0.0310*	
H13B	0.82440	0.53410	0.19310	0.0310*	
H14A	0.92100	0.19040	0.26500	0.0320*	
H14B	0.98570	0.38940	0.24850	0.0320*	
H15A	0.96940	0.33340	0.11390	0.0280*	
H15B	1.02240	0.14110	0.15290	0.0280*	
H16A	0.87490	0.05300	0.06320	0.0250*	
H16B	0.84820	−0.01880	0.14760	0.0250*	

### Atomic displacement parameters (Å^2^)

**Table:**

	*U*^11^	*U*^22^	*U*^33^	*U*^12^	*U*^13^	*U*^23^
N1	0.0151 (12)	0.0199 (13)	0.0168 (13)	−0.0004 (10)	−0.0007 (10)	0.0011 (10)
N2	0.0133 (11)	0.0187 (12)	0.0183 (13)	0.0017 (10)	0.0011 (9)	−0.0014 (10)
N3	0.0146 (12)	0.0250 (13)	0.0179 (13)	0.0045 (10)	0.0004 (10)	−0.0054 (11)
N4	0.0255 (14)	0.0450 (17)	0.0230 (15)	0.0032 (13)	−0.0010 (12)	−0.0044 (13)
N5	0.0203 (14)	0.0315 (15)	0.0267 (15)	0.0043 (12)	0.0014 (11)	−0.0003 (12)
C1	0.0169 (14)	0.0124 (15)	0.0213 (16)	−0.0017 (11)	0.0012 (12)	0.0013 (11)
C2	0.0158 (14)	0.0143 (15)	0.0146 (14)	−0.0008 (12)	−0.0030 (11)	0.0021 (11)
C3	0.0235 (15)	0.0159 (15)	0.0149 (15)	−0.0011 (13)	0.0049 (12)	−0.0010 (12)
C4	0.0178 (15)	0.0158 (15)	0.0166 (15)	−0.0001 (13)	0.0006 (12)	0.0003 (12)
C5	0.0144 (14)	0.0155 (15)	0.0161 (15)	−0.0038 (12)	0.0009 (11)	−0.0011 (11)
C6	0.0185 (15)	0.0262 (16)	0.0183 (15)	0.0012 (13)	0.0038 (13)	0.0002 (13)
C7	0.0212 (15)	0.0182 (15)	0.0126 (15)	0.0034 (13)	−0.0012 (12)	0.0007 (12)
C8	0.0187 (15)	0.0206 (16)	0.0227 (16)	0.0008 (13)	−0.0016 (12)	0.0012 (13)
C9	0.0162 (15)	0.0227 (16)	0.0229 (17)	−0.0004 (13)	0.0025 (12)	−0.0010 (13)
C10	0.0185 (15)	0.0186 (16)	0.0231 (16)	−0.0027 (12)	0.0065 (12)	0.0003 (13)
C11	0.0198 (15)	0.0214 (15)	0.0160 (15)	−0.0013 (13)	0.0003 (12)	−0.0010 (13)
C12	0.0184 (15)	0.0321 (17)	0.0243 (17)	−0.0003 (14)	0.0000 (12)	−0.0095 (14)
C13	0.0227 (17)	0.0302 (18)	0.0233 (17)	0.0015 (14)	−0.0017 (13)	−0.0052 (14)
C14	0.0215 (16)	0.0304 (18)	0.0268 (17)	−0.0030 (14)	−0.0022 (13)	−0.0068 (15)
C15	0.0157 (15)	0.0266 (17)	0.0278 (17)	−0.0013 (13)	0.0019 (12)	−0.0004 (14)
C16	0.0186 (15)	0.0251 (16)	0.0199 (17)	0.0039 (13)	0.0016 (13)	−0.0002 (12)

### Geometric parameters (Å, º)

**Table:**

N1—C1	1.324 (3)	C12—C13	1.521 (4)
N1—C5	1.348 (3)	C13—C14	1.520 (3)
N2—C1	1.406 (3)	C14—C15	1.529 (4)
N2—C8	1.399 (3)	C15—C16	1.519 (4)
N2—C11	1.392 (4)	C3—H3	0.9300
N3—C5	1.349 (3)	C8—H8	0.9300
N3—C12	1.470 (3)	C9—H9	0.9300
N3—C16	1.465 (3)	C10—H10	0.9300
N4—C6	1.153 (4)	C11—H11	0.9300
N5—C7	1.152 (3)	C12—H12A	0.9700
C1—C2	1.403 (4)	C12—H12B	0.9700
C2—C3	1.400 (3)	C13—H13A	0.9700
C2—C6	1.433 (4)	C13—H13B	0.9700
C3—C4	1.383 (4)	C14—H14A	0.9700
C4—C5	1.426 (4)	C14—H14B	0.9700
C4—C7	1.429 (3)	C15—H15A	0.9700
C8—C9	1.351 (4)	C15—H15B	0.9700
C9—C10	1.423 (4)	C16—H16A	0.9700
C10—C11	1.354 (3)	C16—H16B	0.9700
			
C1—N1—C5	121.1 (2)	N2—C8—H8	126.00
C1—N2—C8	128.8 (2)	C9—C8—H8	126.00
C1—N2—C11	123.5 (2)	C8—C9—H9	126.00
C8—N2—C11	107.6 (2)	C10—C9—H9	126.00
C5—N3—C12	121.4 (2)	C9—C10—H10	126.00
C5—N3—C16	123.7 (2)	C11—C10—H10	126.00
C12—N3—C16	113.3 (2)	N2—C11—H11	126.00
N1—C1—N2	113.5 (2)	C10—C11—H11	126.00
N1—C1—C2	122.6 (2)	N3—C12—H12A	110.00
N2—C1—C2	124.0 (2)	N3—C12—H12B	110.00
C1—C2—C3	116.6 (2)	C13—C12—H12A	110.00
C1—C2—C6	127.2 (2)	C13—C12—H12B	110.00
C3—C2—C6	116.3 (2)	H12A—C12—H12B	108.00
C2—C3—C4	121.7 (2)	C12—C13—H13A	109.00
C3—C4—C5	117.5 (2)	C12—C13—H13B	109.00
C3—C4—C7	117.5 (2)	C14—C13—H13A	109.00
C5—C4—C7	124.6 (2)	C14—C13—H13B	109.00
N1—C5—N3	116.8 (2)	H13A—C13—H13B	108.00
N1—C5—C4	120.2 (2)	C13—C14—H14A	110.00
N3—C5—C4	123.0 (2)	C13—C14—H14B	110.00
N4—C6—C2	177.1 (3)	C15—C14—H14A	110.00
N5—C7—C4	178.0 (3)	C15—C14—H14B	110.00
N2—C8—C9	108.3 (2)	H14A—C14—H14B	108.00
C8—C9—C10	107.8 (2)	C14—C15—H15A	110.00
C9—C10—C11	107.9 (2)	C14—C15—H15B	110.00
N2—C11—C10	108.3 (2)	C16—C15—H15A	110.00
N3—C12—C13	108.9 (2)	C16—C15—H15B	110.00
C12—C13—C14	111.7 (2)	H15A—C15—H15B	108.00
C13—C14—C15	110.5 (2)	N3—C16—H16A	110.00
C14—C15—C16	109.5 (2)	N3—C16—H16B	110.00
N3—C16—C15	110.5 (2)	C15—C16—H16A	110.00
C2—C3—H3	119.00	C15—C16—H16B	110.00
C4—C3—H3	119.00	H16A—C16—H16B	108.00
			
C5—N1—C1—N2	177.4 (2)	N2—C1—C2—C3	178.3 (2)
C5—N1—C1—C2	−1.4 (4)	N2—C1—C2—C6	−1.0 (4)
C1—N1—C5—N3	−177.0 (2)	N1—C1—C2—C6	177.7 (3)
C1—N1—C5—C4	5.8 (4)	N1—C1—C2—C3	−3.0 (4)
C8—N2—C1—N1	178.4 (2)	C1—C2—C3—C4	3.0 (4)
C11—N2—C1—C2	174.0 (2)	C6—C2—C3—C4	−177.6 (3)
C1—N2—C8—C9	177.1 (2)	C2—C3—C4—C7	−171.7 (3)
C1—N2—C11—C10	−177.3 (2)	C2—C3—C4—C5	1.1 (4)
C8—N2—C11—C10	0.0 (3)	C3—C4—C5—N1	−5.5 (4)
C8—N2—C1—C2	−2.8 (4)	C7—C4—C5—N3	−10.5 (4)
C11—N2—C8—C9	0.0 (3)	C3—C4—C5—N3	177.4 (2)
C11—N2—C1—N1	−4.8 (3)	C7—C4—C5—N1	166.6 (2)
C5—N3—C16—C15	134.0 (2)	N2—C8—C9—C10	0.0 (3)
C12—N3—C5—N1	−14.1 (3)	C8—C9—C10—C11	0.0 (3)
C12—N3—C16—C15	−60.3 (3)	C9—C10—C11—N2	0.0 (3)
C16—N3—C5—C4	−32.4 (4)	N3—C12—C13—C14	−55.5 (3)
C5—N3—C12—C13	−135.4 (2)	C12—C13—C14—C15	55.1 (3)
C16—N3—C12—C13	58.5 (3)	C13—C14—C15—C16	−54.7 (3)
C16—N3—C5—N1	150.5 (2)	C14—C15—C16—N3	56.7 (3)
C12—N3—C5—C4	163.0 (2)		

### Hydrogen-bond geometry (Å, º)

**Table:**

*D*—H···*A*	*D*—H	H···*A*	*D*···*A*	*D*—H···*A*
C12—H12*B*···N1	0.97	2.36	2.740 (3)	103
